# Patient Perception of Vocal Tremor Severity and its Relationship to Acoustic Voice Outcomes: An Exploratory Study

**DOI:** 10.5334/tohm.1087

**Published:** 2025-12-04

**Authors:** James C. Borders, Gary Gartling, Elizabeth Hary, Milan R. Amin, Michelle S. Troche, Julie Barkmeier-Kraemer, Ryan C. Branski

**Affiliations:** 1 Department of Speech, Language, and Hearing Sciences, Boston University, Boston, MA, US; 2 Otolaryngology-Head & Neck Surgery, NYU Grossman School of Medicine, New York, NY, US; 3 Department of Communication Science and Disorders, School of Health and Rehabilitation Sciences, University of Pittsburgh, Pittsburgh, PA, US; 4 Laboratory for the Study of Upper Airway Dysfunction, Department of Biobehavioral Sciences, Teachers College, Columbia University, New York, NY, US; 5 Otolaryngology-Head & Neck Surgery, University of Utah, Salt Lake City, UT, US; 6 Communication Sciences & Disorders, University of Utah, Salt Lake City, UT, US

**Keywords:** Vocal Tremor, Dysphonia, Patient Perception

## Abstract

**Background:**

Vocal tremor profoundly impacts communication, social participation, and quality of life. Although expert auditory-perceptual ratings of vocal tremor severity align with acoustic voice outcomes (e.g., extent of frequency (*f*_o_) and intensity modulation), patient perception of their voice remains unexamined despite its clinical importance. This study aimed to characterize the relationship between patient-reported vocal tremor severity and acoustic voice outcomes at baseline and after botulinum toxin injections.

**Method:**

Patients diagnosed with vocal tremor affecting multiple structures (ETvt) or tremor only observed in the larynx (LDvt) were recruited. Participants completed the voice section of the Quality of Life in Essential Tremor questionnaire to assess patient perception and performed sustained /ɑ/ at a comfortable pitch and volume, from which acoustic voice outcomes (rate and extent of fundamental frequency [*f_o_*] and amplitude [dB] modulation) were derived. A subset of participants received botulinum toxin injections and were reassessed within the therapeutic window (within 12 weeks).

**Results:**

Thirty participants (29 females; mean age = 72 years, SD = 11.40) were analyzed. Participants who rated their vocal tremor as “severe” demonstrated higher rate *f_o_* (β = 1.20, 95% CI: –0.10, 2.60 Hz) and rate dB (β = 2.30, 95% CI: 0.50, 4.10 Hz) compared to participants who rated their tremor as “moderate”. Participants who rated their tremor as “marked” demonstrated higher rate *f_o_* (β = 1.50, 95% CI: 0.30, 2.60 Hz) compared to “moderate” ratings. Improvements in patient perception of vocal tremor and acoustic outcomes were highly heterogenous among seven participants who received botulinum toxin.

**Discussion:**

Participants reporting more severe vocal tremor demonstrated more aberrant acoustic voice outcomes. After botulinum toxin injection, substantial heterogeneity was observed in acoustic voice measures which varied based on patient perception of change. These preliminary, exploratory findings provide a foundation for future investigations to define meaningful change in this population.

## Introduction

Vocal tremor is characterized by nearly rhythmic voice shakiness resulting from involuntary modulation of pitch and loudness and most commonly associated with movement disorders such as essential tremor, laryngeal dystonia, and Parkinson’s disease [1,2]. These voice disruptions can profoundly impact communication, limit social participation, and reduce quality of life [3]. Among individuals with vocal tremor, otolaryngologists and speech pathologists routinely evaluate changes in voice function using various auditory-perceptual, acoustic, and patient-reported outcome measures [4]. Although comprehensive voice evaluation necessitates some degree of objective assessment, a patient’s perspective of their voice is of paramount importance [5]. For example, a potential clinical scenario is one in which acoustic measures show improvement following treatment, yet the patient reports no improvement in their voice.

To date, *expert* ratings of vocal tremor severity (e.g., speech pathologists, otolaryngologists), rather than *patient* perception, are commonly employed and related to quantitative acoustic outcomes [2,6,7,8,9,10]. Two methodological approaches have been used to investigate this relationship among *expert* raters: (1) acoustic analyses of natural speech samples [6,10] and (2) computational models using synthetic modulation of acoustic parameters [2,7,8,9]. Overall, findings suggest that auditory-perceptual judgments of vocal tremor severity are influenced by the *extent* of fundamental frequency (*f_o_*) and intensity (i.e., amplitude) modulation [2,6,7,8,9]. Furthermore, the *rate* of tremor modulation, particularly in the presence of large amplitudes, may negatively affect auditory-perceptual judgments [8], whereas others have not found a relationship and instead suggested that tremor rate may interact with other acoustic parameters in a nonlinear manner [6,9]. In addition to these acoustic features, variation in their perceptual correlates, namely pitch and loudness, may also shape expert ratings [10]. Despite this growing body of literature, it remains unclear how these variables align with the *patient’s* perception of their own voice.

Although botulinum toxin injection is the most common treatment for vocal tremor, reported success rates (i.e., improvements in acoustic and/or perceptual outcomes) may be as low as 50% [11,12,13]. Across studies, botulinum toxin injection has been associated with reduced modulation of *f_o_* [11,12]. However, far less is known about how patients perceive changes in their voice following treatment, or how these subjective impressions correspond to objective acoustic measures. Adler et al. (2004) reported an average improvement of 1.70 points on a 5-point tremor severity scale six weeks after injection [13]. Additionally, Hertegard, Garnqvist, and Lindestad (2000) found that 67% of participants rated the treatment positively; however, these ratings focused on the overall treatment experience, such as side effects, and were not limited to voice-specific outcomes [11]. Understanding how patient perception aligns with acoustic changes following botulinum toxin injection is essential for identifying voice outcomes that are meaningful to patients and for establishing benchmarks for clinically meaningful change.

This prospective, exploratory study aimed to characterize the relationship between patient perception of vocal tremor severity and acoustic voice outcomes. As a secondary aim, we explored how changes in acoustic outcomes align with changes in patient perception in a small subset of individuals who received botulinum toxin injection. Collectively, this work has important clinical implications for understanding the interplay between quality of life, acoustic voice parameters, and patient perception in individuals with vocal tremor.

## Method

### Participants

Participants with vocal tremor associated with the co-morbidity of essential tremor (ETvt) or laryngeal dystonia (LDvt) were recruited. Diagnoses were established by consensus between an otolaryngologist and neurologist following current classification criteria [14]. These determinations were based on a comprehensive history and examination. Inclusion criteria required participants to be between 18 and 90 years of age. Participants with other neurological conditions (e.g., Parkinson’s disease, stroke, etc.) were excluded. This study was approved by the NYU Grossman School of Medicine Institutional Review Board (S21-00005) and conducted at a tertiary laryngology clinic.

### Assessment Procedures & Data Analysis

All participants completed a baseline evaluation, which included the Quality of Life in Essential Tremor Questionnaire (QUEST). The QUEST is a patient-reported outcome measure examining the functional impact of tremor [15]. For the purposes of this investigation, participants rated the severity of their vocal tremor using a five-point scale on the QUEST (QUEST-Voice): None (no tremor at any time), Mild (mild tremor not causing difficulty in performing any activities), Moderate (tremor causes difficulty in performing some activities), Marked (tremor causes difficulty in performing most or all activities), or Severe (tremor prevents performing some activities). Participants also underwent indirect visualization via nasoendoscopy of the speech structures (velum, pharynx, and larynx) at rest and during speech tasks. Tremor location was assessed during nasoendoscopy by an otolaryngologist.

Voice recordings (sustained phonation and connected speech tasks) were collected in a quiet clinic room in accordance with established protocols [7]. Sustained /ɑ/ at comfortable pitch and volume was used for analysis of tremor characteristics. Recordings were made using The Computerized Speech Lab system (CSL Model 4500B, PENTAX Medical) with an AKG P220 microphone positioned on a stand approximately 4 – 6cm from the lips at a 45-degree angle and a sampling rate of 44.1kHz. While efforts were made to standardize the mouth-to-microphone distance, speakers with head tremor may have exhibited variability in this distance. During recordings, participants were instructed to sit upright and avoid head movement. The middle two seconds of each /ɑ/ production were extracted and analyzed using Praat [16]. Semi-automated algorithms were used to calculate the rate of fundamental frequency (*f_o_*) modulation (rate *f_o_*; Hz), the rate of amplitude (intensity) modulation (rate dB; Hz), the extent of *f_o_* modulation (extent *f_o_;* %), and the extent of amplitude modulation (extent dB; %) [17]. For the analysis, segments of sustained /ɑ/ between one to three seconds were extracted. The gold standard for acoustic analysis is to capture the middle two to three seconds of sustained phonation to avoid variability associated with voice onset and offset. However, due to the severity of vocal tremor in some participants, only approximately one second of continuous phonation could be analyzed in some cases [6,18]. The semi-automated analysis combined manual and automated steps. Manually, a 1 – 3 second segment was selected, the minimum and maximum pitch and amplitude was identified, and the upper and lower bound of the *f_o_* range was defined. To ensure accurate pitch tracking, a 10 Hz buffer was applied above and below the identified pitch range, minimizing potential human or software error. The automated script then computed the rate and extent of *f_o_* and dB modulation using these defined parameters. Specifically, this involves calculating the frequency and amplitude contours of the input signal, removing linear trends and normalizing the contours, and then determining vocal tremor indices [17,19].

A subset of seven participants received botulinum toxin injections. These participants repeated the acoustic voice recordings and QUEST within the therapeutic window following injection (within 12 weeks) [20].

### Statistical Analysis

A Bayesian statistical framework, which incorporates prior beliefs, expectations, or evidence through probability distributions on model parameters, was employed. This approach requires specifying a prior distribution that reflects one’s beliefs before observing data, which can range from “highly informative” (i.e., based on effect sizes from prior research) to weakly informative (i.e., restricting implausible values to facilitate model convergence and estimation). The model (i.e., likelihood) produces a posterior distribution, which represents the distribution of plausible parameter values conditioned on the data and prior distribution. The posterior distribution can then be summarized with a point estimate, as well as a credible interval (CI) that represents the probability that a parameter falls within a given range (e.g., 95%) or direction (e.g., a positive or negative treatment effect). Bayesian statistical models included ‘weakly informative’ prior distributions that restricted theoretically or physiologically implausible values **(Appendix A)**.

To characterize the relationship between patient perceptions of vocal tremor severity and acoustic outcomes, a Bayesian linear model with a fixed effect of patient vocal tremor severity rating was employed for each acoustic outcome. Point estimates and 95% CIs of pairwise comparisons were examined from the model, as well as probability of direction (*pd*; i.e., the posterior probability that the difference between ratings is greater than zero). To explore how this relationship differed between participants with tremor affecting multiple structures (ETvt) to those with tremor only observed in the larynx (LDvt), an additional Bayesian linear model with an interaction between patient vocal tremor severity rating and these two diagnostic groups was employed. Considering the small sample size and exploratory nature of this study, descriptive statistics were conducted to evaluate trends between participants who reported positive, negative, and no change in perceptions of vocal tremor severity after treatment. Specifically, a meaningful amount of change was defined as 4Hz based on previous acoustic-perceptual experiments [9]. Additionally, a lower extent and rate of modulation was specified as less aberrant [8]. Analyses were performed in R (v4.2.1) [21] with the *brms* package [22].

## Results

Thirty-five participants were enrolled (32 females, 3 males) with an average age of 71 years (SD = 13.74). Three participants did not complete the QUEST-Voice. Acoustic data could not be extracted for two participants due to severe voice breaks. Semi-automated analysis script was unable to derive rate dB or extent dB for three additional participants due to imperceptible changes in amplitude and was unable to derive rate *f_o_* or extent *f_o_* for one participant due to severe voice breaks. The final data set included 30 participants (29 female, 1 male) with an average age of 72 years (SD = 11.40). Eighteen of these participants had ETvt and 12 had LDvt. Four of these participants reported head tremor. Five participants rated their voice as “severe”, ten as “marked”, twelve as “moderate”, one as “mild”, and two as “none”. Due to the low number of “none” and “mild” ratings, these participants were excluded from statistical models.

### Relationship between patient perception of vocal tremor severity and acoustic outcomes

#### Rate of fundamental frequency (*f_o_*) modulation (rate *f_o_*)

**Figure 1:**
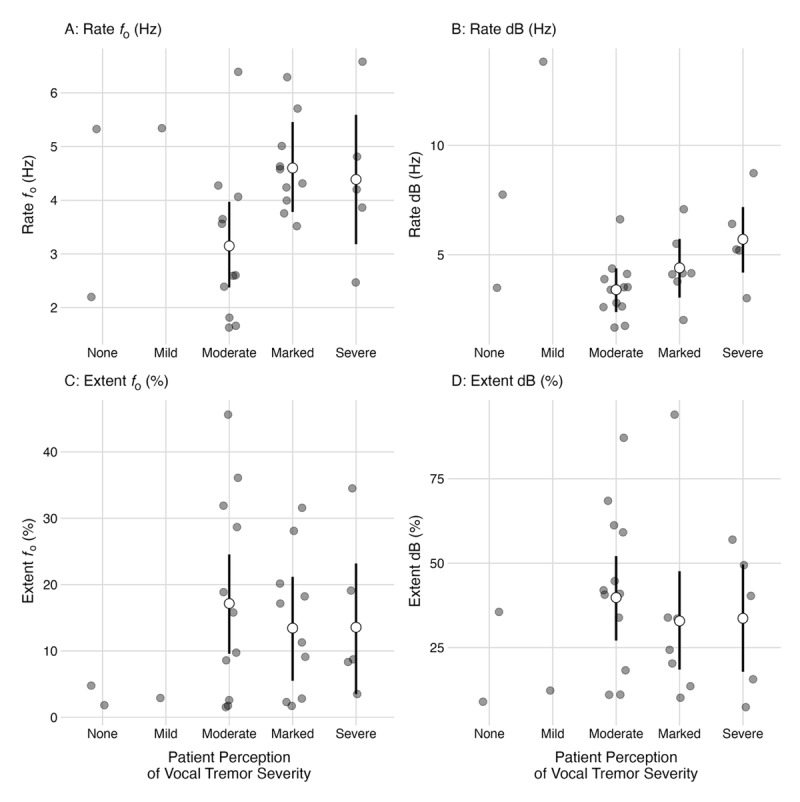
Relationship between patient perceptions of vocal tremor severity and acoustic outcomes. *Caption*: Raw data points are shown with the point estimate and 95% credible interval from the statistical model across acoustic outcomes. Rate *f_o_* refers to the rate of fundamental frequency (*f_o_*) modulation. Rate dB refers to the rate of amplitude (dB) modulation. Extent *f_o_* refers to the extent of fundamental frequency (*f_o_*) modulation. Extent dB refers to the extent (dB) of amplitude modulation.

Among participants who rated their voice as “severe”, no meaningful differences emerged compared to participants who rated their voice as “marked” (β = –0.20, 95% CI: –1.60, 1.10; *pd* = 62%). Among participants who rated their voice as “severe”, a 1.20Hz higher rate *f_o_* (95% CI: –0.10, 2.60 Hz) was observed compared to participants who rated their voice as “moderate” (Figure 1). The posterior probability this difference was greater than zero was 96.50%. Among participants who rated their voice as “marked”, a 1.50Hz higher rate *f_o_* was observed compared to participants who rated their voice “moderate” (95% CI: 0.30, 2.60). The posterior probability this difference was greater than zero was 99.70%.

**Figure 2:**
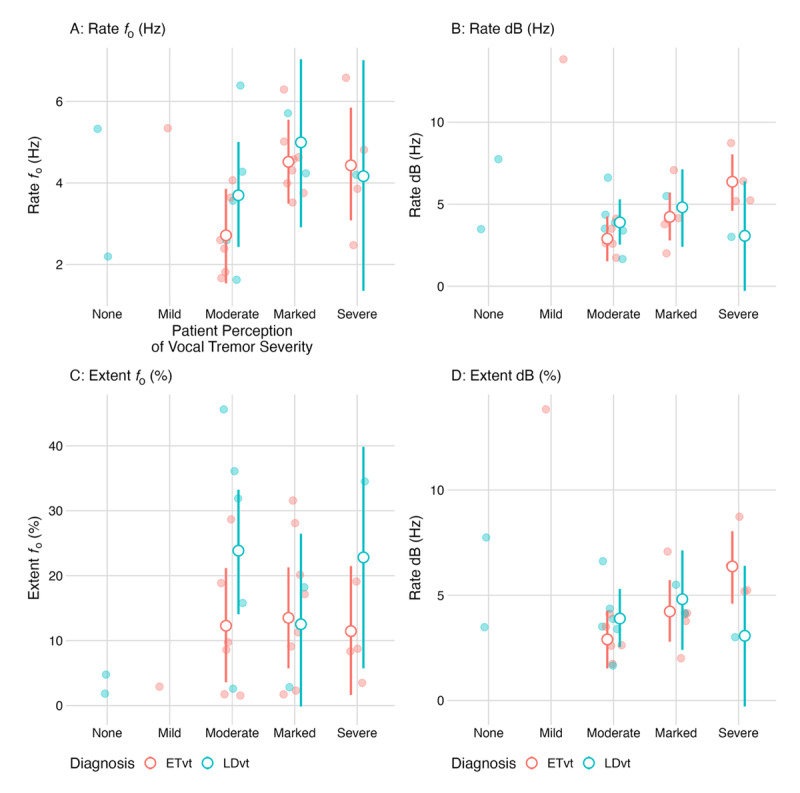
Relationship between patient perceptions of vocal tremor severity and acoustic outcomes across diagnoses. *Caption*: Raw data points are shown with the point estimate and 95% credible interval from the statistical model across acoustic outcomes. Different colors represent diagnosis sub-groups, specifically essential tremor related vocal tremor (ETvt) in pink and laryngeal dystonia (LDvt) in blue. Rate *f_o_* refers to the rate of fundamental frequency (*f_o_*) modulation. Rate dB refers to the rate of amplitude (dB) modulation. Extent *f_o_* refers to the extent of fundamental frequency (*f_o_*) modulation. Extent dB refers to the extent (dB) of amplitude modulation.

Among individuals with ETvt, participants who rated their voice as “marked” demonstrated a 1.81Hz higher rate *f_o_* (95% CI: 0.30, 3.30 Hz) compared to ETvt participants who rated their voice as “moderate” (*pd* = 99%; Figure 2). No meaningful differences in rate *f_o_* were observed between diagnoses (i.e., ETvt vs. LDvt) across any level of perceptual vocal tremor severity.

#### Rate of amplitude modulation (rate dB)

Among participants who rated their voice as “severe”, no meaningful differences emerged compared to participants who rated their voice as “marked” (β = 1.30, 95% CI: –0.70, 3.30; *pd* = 91%). Among participants who rated their voice as “severe”, a 2.30 Hz higher rate dB was observed compared to participants who rated their voice as “moderate” (95% CI: 0.50, 4.10). The posterior probability this difference was greater than zero was 99%. Among participants who rated their voice as “marked”, no meaningful differences emerged compared to participants who rated their voice as “moderate” (β = 1.00, 95% CI: –0.60, 2.60; *pd* = 87%).

Among individuals with ETvt, participants who rated their voice as “severe” demonstrated a higher rate dB modulation compared to participants who rated their voice as “marked” (β = 2.10, 95% CI: –0.20, 4.30; *pd* = 96%) and “moderate” (β = 3.50, 95% CI: 1.20, 5.70; *pd* = 99%). No meaningful differences in rate dB were observed between diagnoses across any level of perceptual vocal tremor severity.

#### Extent of fundamental frequency (fo) modulation (extent fo)

Among participants who rated their voice as “severe”, no meaningful differences emerged compared to participants who rated their voice as “marked” (β = 0.20, 95% CI: –12.30, 12.30; *pd* = 51%). Among participants who rated their voice as “marked”, no meaningful differences were observed in extent *f_o_* compared to participants who rated their voice as either “moderate” (Δ = –3.70, 95% CI: –14.40, 6.90; *pd* = 76%) or “severe” (β = –3.60, 95% CI: –15.70, 8.20; *pd* = 72%).

Within each diagnosis, no meaningful differences were observed between vocal tremor severity ratings. Between diagnoses, individuals with LDvt who rated their voice as “moderate”, demonstrated higher extent *f_o_* modulation compared to ETvt participants rating their voice as “moderate” (β = 11.50, 95% CI: –0.50, 24.14; *pd* = 97%).

#### Extent of amplitude modulation (extent dB)

Among participants who rated their voice as “severe”, no meaningful difference emerged compared to participants who rated their voice “marked” (β = 0.50, 95% CI: –18, 20; *pd* = 52%). Among participants who rated their voice as “marked”, no meaningful differences in extent dB were observed compared to those participants who rated their voice as either “moderate” (β = –6.90, 95% CI: –24, 10.90; *pd* = 79%) or “severe” (β = –6.30, 95% CI: –24, 11.90; *pd* = 75%). There were no meaningful differences for extent dB modulation between diagnoses.

### Characterization of patient perceptions of vocal tremor severity and acoustic outcomes after botulinum toxin injection

Among seven participants who received botulinum toxin injections, three received injection to the thyroarytenoid muscles, two to the false vocal folds to target the thyroarytenoids, one to the suprahyoid strap muscles, and one to both the thyroarytenoids and suprahyoids (Table 1). The average time from injection to voice assessment was 35 days (SD = 14; range = 24 – 61). Three participants (IDs 1, 3, and 5) reported improved vocal tremor severity (Table 2), which aligned with improved rate dB for ID 1 (4.20 Hz) and ID 5 (4.50 Hz), and both extent dB (14.10%) and extent *f_o_* (23.80%) for ID 3.

**Table 1:** Description of tremor distribution, treatment and participant characteristics.


ID	AGE	SEX	CONSENSUS DIAGNOSIS	BOTULINUM TOXIN DOSE

1	75	Female	ETvt	7 units each false vocal fold

2	68	Female	LDvt	3.5 units each thyroarytenoid

3	84	Female	ETvt	3.0 units each supra-hyoid strap

4	73	Female	ETvt	2.5 units each false vocal fold

5	56	Female	LDvt	0.75 units left thyroarytenoid; 1-unit right thyroarytenoid

6	79	Female	ETvt	0.8 units each thyroarytenoid

7	78	Female	ETvt	1.0 unit each thyroarytenoid; 1.5 units each supra-hyoid strap

8	70	Male	LDvt	Not applicable.

9	78	Female	LDvt	

10	57	Male	ETvt	

11	67	Female	ETvt	

12	75	Female	ETvt	

13	76	Female	ETvt	

14	69	Female	ETvt	

15	73	Female	ETvt	

16	71	Female	LDvt	

17	82	Female	LDvt	

18	62	Female	LDvt	

19	76	Female	ETvt	

20	26	Female	ETvt	

21	75	Female	ETvt	

22	63	Female	ETvt	

23	73	Female	LDvt	

24	74	Female	ETvt	

25	71	Female	ETvt	

26	75	Female	ETvt	

27	83	Female	LDvt	

28	87	Female	LDvt	

29	83	Female	LDvt	

30	73	Female	LDvt	


ETvt: essential tremor-related vocal tremor. LDvt: Laryngeal dystonia-associated vocal tremor.

**Table 2:** Description of tremor characteristics across participants.


ID	BOTULINUM TOXIN DOSE	CHANGE IN PATIENT PERCEPTION		MEAN CHANGE IN ACOUSTIC OUTCOME FROM PRE- TO POST- BOTULINUM TOXIN INJECTION			

				RATE *f_O_*	EXTENT *f_O_*	RATE DB	EXTENT DB

1	7 units each false vocal fold	Severe → Marked	Positive	0	0	4.20*	–4.40
	
3	3.0 units each supra-hyoid strap	Severe → Marked		1	23.80*	–0.20	14.10*
	
4	2.5 units each false vocal fold	Severe → Marked		0.20	3.40	0.23	–6.30
	
5	0.75 units left thyroarytenoid; 1-unit right thyroarytenoid	Moderate → Mild		2.30	–7.50	4.50*	–28.40

2	3.5 units each thyroarytenoid	Severe → Severe	No Change	–2.38	4.30	–0.40	25.20
	
6	0.8 units each thyroarytenoid	Marked → Marked		1	0.10	–0.40	–22.40

7	1.0 unit each thyroarytenoid; 1.5 units each supra-hyoid strap	Marked → Severe	Negative	–0.70	8	–1.60	–7.80^+^


*Caption:* Mean change is pre minus post, where positive values indicate improvement. * indicates improved acoustic outcome that aligns with patient perception. ^+^ indicates worsened acoustic outcome that aligns with patient perception.

Two participants (IDs 2 and 6) reported no change in vocal tremor severity. However, participant 2 showed a large improvement in extent dB (25.20%). One participant (ID 7) reported worsening of vocal tremor severity after injection, which corresponded with a decline in extent dB (–7.80%).

## Discussion

To date, research on vocal tremor has primarily focused on expert auditory-perceptual ratings and acoustic analyses, despite the critical importance of patient experience and perception of their voice. This exploratory study provides preliminary insight into the relationship between patient-reported vocal tremor severity and acoustic measures. In addition, we investigated this relationship before and after treatment for a small group of patients who elected to receive botulinum toxin injections. Acoustic measures of the rate of fundamental frequency (Rate *f_o_*) and amplitude modulation (Rate dB) most closely aligned with patient perception of vocal tremor severity. These findings suggest modulation rate, rather than extent, may more accurately reflect the perceptual impact of tremor, underscoring their potential value in clinical assessment and treatment monitoring. Following botulinum toxin injection, improved rate of *f_o_* modulation (rate *f_o_*) as well as rate and extent of intensity modulation (extent dB) aligned with improved patient perceptions of their voice, though changes in acoustic outcomes were highly variable in this small, heterogeneous cohort. These findings represent an important first step toward elucidating the complex interplay between quality of life, acoustic voice outcomes, and patient perceptions of vocal tremor.

This study addresses a key gap in the literature regarding patient perceptions of vocal tremor severity. This work extends prior studies by demonstrating rate of fundamental frequency (Rate *f*_o_), which has previously been shown to influence expert ratings [6,8], as well as rate of amplitude modulation (Rate dB) are associated with patient perceptions of their voice. The magnitude of these relationships was modest, likely due in part to the use of an ordinal scale which is less sensitive to differences compared to continuous measures (e.g., visual analog scale). Furthermore, the relationship between acoustic outcomes and patient perception was dependent on diagnosis (i.e., LDvt compared to ETvt). Together, these findings suggest changes in the rate of frequency and amplitude modulation may serve as important acoustic outcomes to establish meaningful change for patients and sensitively track changes in voice function.

Although botulinum toxin injection is a common treatment for vocal tremor, efficacy remains highly variable. This variability may be attributed to heterogeneity in vocal tremor presentations or differences in patient perceived voice changes. Notably, four participants reported subjective improvement in vocal tremor after botulinum toxin injection, which corresponded with improved extent of intensity modulation (Extent dB). However, the magnitude of these acoustic changes was highly variable, possibly reflecting variability in clinical presentations, injection site, and/or dosing.

Several limitations should be acknowledged. First, participants with mild vocal tremor were excluded due to a very low number in our sample. Some participants with severe vocal tremor were also excluded from acoustic analysis due to substantial voice breaks which limited analysis by semi-automated algorithms. This exclusion of mild and severe participants likely introduced selection bias, limiting the generalizability of our findings. Acoustic outcomes (e.g., tremor rate, regularity, amplitude) may also interact with auditory-perceptual ratings [8]. However, our sample size was insufficient to examine these interactions. This interaction will be an important area of future investigation. Our cohort was also substantively heterogeneous with regard to tremor location across upper airway structures and vocal tremor phenotypes, which influenced group-level estimates. Future research in larger cohorts is needed to determine how tremor location impacts both objective voice measures and patient subjective voice experience. Many participants had previously received botulinum toxin injection prior to study enrollment, which may have affected treatment outcomes and patient perceptions. Finally, although efforts were made to standardize the mouth-to-microphone distance during acoustic recordings, participants with head tremor may have exhibited greater variability, potentially affecting the accuracy of intensity modulation measurements.

## Conclusions

The patient’s experience and perception of their voice is of paramount importance. However, research on vocal tremor has largely focused on *expert* auditory-perceptual ratings in the context of acoustic voice changes. Findings from this exploratory study suggest that acoustic measures of the rate of fundamental frequency (Rate *f_o_*) and amplitude modulation (Rate dB) most closely aligned with patient perception of vocal tremor severity. These results are limited by the small sample size and substantial heterogeneity related to vocal tremor phenotypes. Future research should continue to investigate these relationships in a longitudinal manner with the goal of defining meaningful change for patients with vocal tremor to sensitively track changes in voice function from an intervention or natural disease process.

## Data Accessibility Statement

Participants of this study did not provide written consent for their data to be shared publicly, so due to the sensitive nature of the research supporting data is not available.

## Figures and Tables

**Figure:**
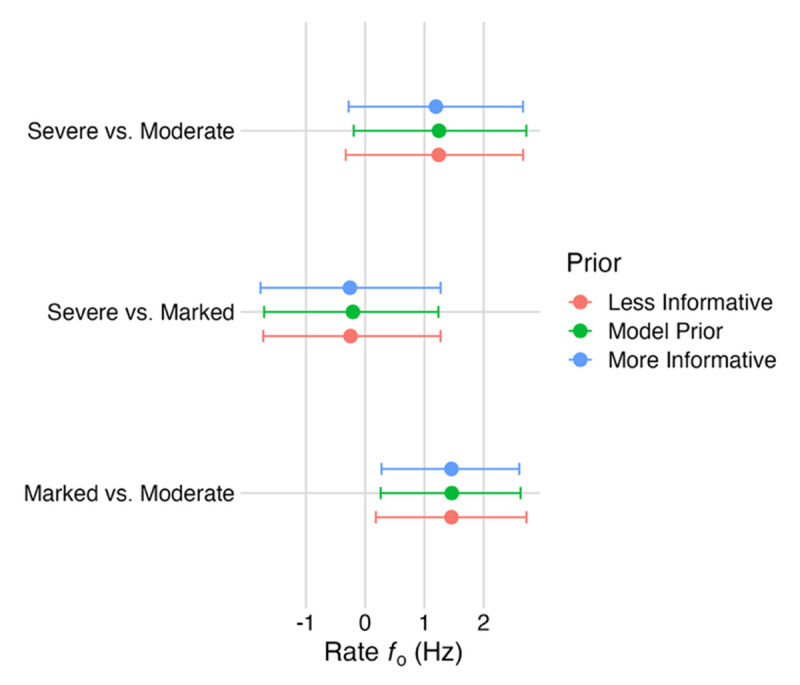
Rate of fundamental frequency (f_o_) modulation

**Figure:**
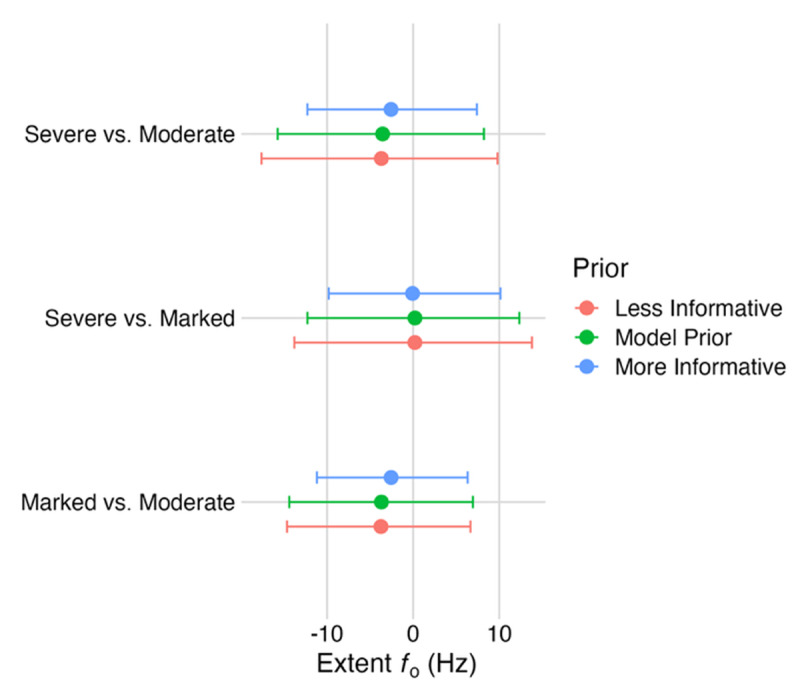
Extent of fundamental frequency (f_o_) modulation

**Figure:**
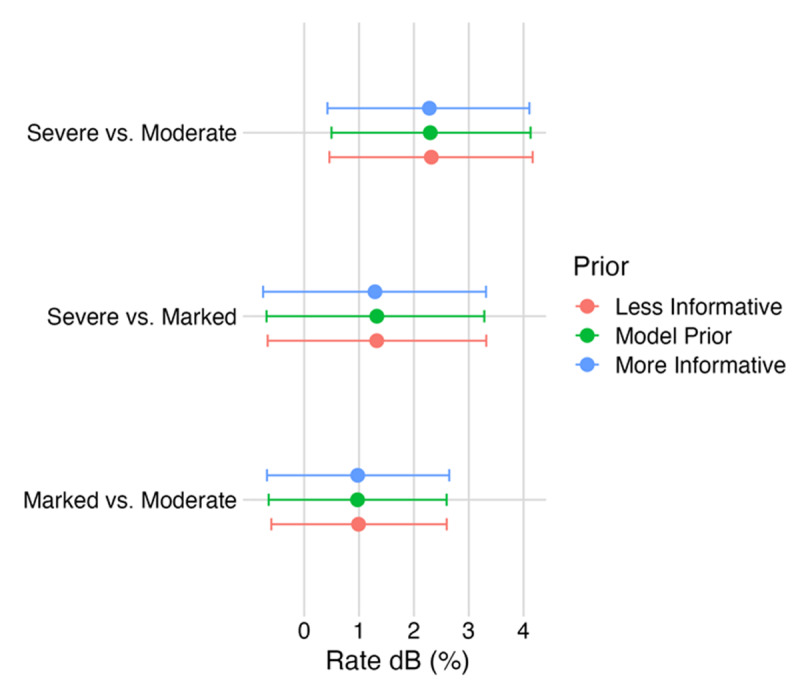
Rate of amplitude modulation (dB)

**Figure:**
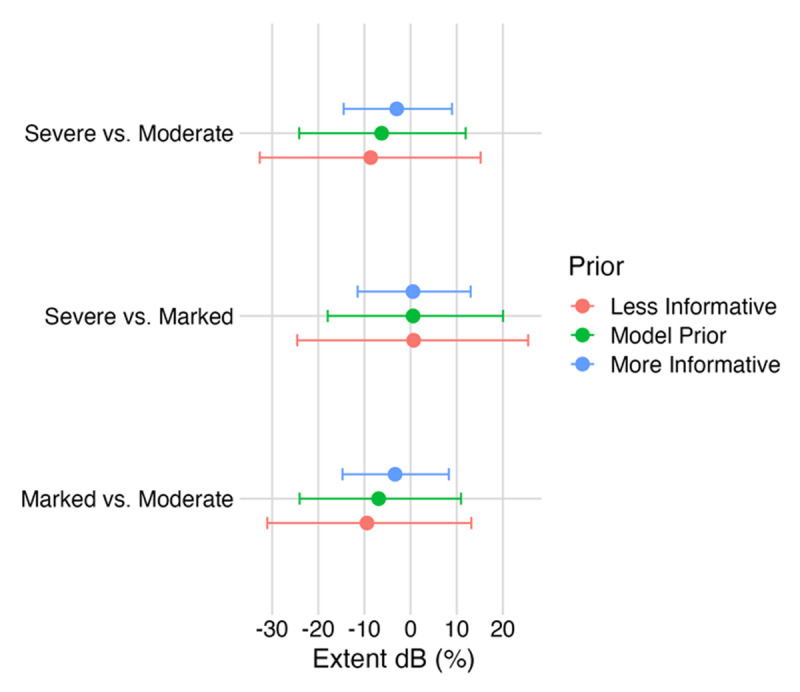
Extent of amplitude modulation (dB)

## Appendix A: Description of Bayesian prior distributions and alternative prior specifications

“Weakly informative” prior distributions were used to constrain implausible values without assuming an *a priori* effect. For each model, we specified prior distributions for the fixed effect. Given the model structure and variables were consistent across all models, the same prior distributions were applied throughout. The prior distribution was specified as a normal distribution centered at zero (assuming no a priori effect) with a standard deviation of 10. This places 95% of the prior probability within ± 20 on the outcome scale, representing plausible differences between patient perception categories while helping to stabilize estimation in the context of a relatively small sample.

To assess the impact of prior distributions on these results, two alternative prior distributions were specified: “more informative” and “less informative” prior distributions. The “more informative” prior distribution was a normal distribution centered at zero with a standard deviation of 5, whereas the “less informative” prior distribution was a normal distribution centered at zero with a standard deviation of 20. Results displayed in the figures below demonstrate that results were generally robust to the choice of prior distribution. However, the choice of prior distribution did impact the precision of model estimates for two outcomes (extent *f_o_* and extent dB).
